# Triangular test design to evaluate tinidazole in the prevention of *Plasmodium vivax* relapse

**DOI:** 10.1186/1475-2875-12-173

**Published:** 2013-05-29

**Authors:** Louis Macareo, Khin Maung Lwin, Phaik Yeong Cheah, Prayoon Yuentrakul, R Scott Miller, Francois Nosten

**Affiliations:** 1 Walter Reed Army Institute of Research, 503 Robert Grant Avenue, Silver Spring, MD 20910, USA; 2 Shoklo Malaria Research Unit, 68/30 Bantung Road, PO Box 46, Mae Sod 63110, Thailand; 3 Mahidol Oxford Tropical Medicine Research Unit, Faculty of Tropical Medicine, Mahidol University, 420/6 Rajvithi Rd, Ratchathewi, Bangkok 10400, Thailand; 4 Centre for Tropical Medicine, Nuffield Department of Clinical Medicine, CCVTM, University of Oxford, Oxford OX3 7LJ, UK

**Keywords:** Malaria, *Plasmodium vivax*, Tinidazole, Relapse, Triangular test, Sequential analysis

## Abstract

**Background:**

There are very few drugs that prevent the relapse of *Plasmodium vivax* malaria in man. Tinidazole is a 5-nitroimidazole approved in the USA for the treatment of indications including amoebiasis and giardiasis. In the non-human primate relapsing *Plasmodium cynomolgi*/macaque malaria model, tinidazole cured one of six macaques studied with an apparent mild delay to relapse in the other five of 14–28 days compared to 11–12 days in controls. One study has demonstrated activity against *P. vivax* in man. Presented here are the results of a pilot phase II, randomized, open-label study conducted along the Thai-Myanmar border designed to evaluate the efficacy of tinidazole to prevent relapse of *P. vivax* when administered with chloroquine*.*

**Methods:**

This study utilized a modified triangular test sequential analysis which allows repeated statistical evaluation during the course of enrolment while maintaining a specified power and type 1 error and minimizing recruitment of subjects. Enrolment was to be halted when a pre-specified success/failure ratio was surpassed. The study was designed to have a 5% type 1 error and 90% power to show whether tinidazole would produce a relapse rate of less than 20% or greater than 45% through Day 63 of weekly follow-up after initiation of treatment and initial parasite clearance with 3 days of an oral weight based dosing of chloroquine and five days of 2 grams/day of tinidazole.

**Results:**

All subjects cleared their parasitaemia by Day 3. Six of the first seven subjects treated with tinidazole relapsed prior to Day 63 (average Day 48.3 (range 42–56)). This exceeded the upper boundary of the triangular test and enrolment to receive tinidazole was halted. A concurrent cohort of five subjects definitively treated with standard doses of primaquine and chloroquine (historically 100% effective) showed no episodes of recurrent *P. vivax* parasitaemia during the 63-day protocol specified follow-up period.

**Conclusions:**

Tinidazole is ineffective in preventing relapse of *P. vivax* at the dose used. The macaque relapsing model appeared to correctly predict outcome in humans. Use of the modified triangular test allowed minimal enrolment and limited unnecessary exposure to the study drug and reduced costs. This adds weight to the ethical and economic advantages of this study design to evaluate similarly situated drugs.

**Trial registration:**

ClinicalTrials.gov NCT00811096

## Background

*Plasmodium vivax* accounts for 40% of malaria and is one of the forms of relapsing malaria due to a persistent liver stage of the disease in the form of hypnozoites. There is presently only one approved treatment (primaquine 15-30 mg base/d for 14 days) that adequately eliminates the persistent liver stage of *P. vivax,* achieving what is referred to as “radical cure” when used in combination with a blood-stage anti-malarial agent. Primaquine and other members of its 8-aminoquinoline class cause dose-dependent oxidative haemolysis, particularly in G6PD (glucose-6-phosphate-dehydrogenase) deficient individuals and requires 14 day dosing leading to problems with compliance [1-4]. Therefore, the use of primaquine requires medical supervision and pre-treatment testing for G6PD deficiency, especially in areas where severe deficient phenotypes are known to occur, thus markedly limiting the utility of primaquine for use in large scale malaria control and elimination programmes. It is important to discover new drugs with an acceptable safety and tolerability profile, with a shorter course of treatment that can eliminate the persistent liver stage of *P. vivax,* thus improving compliance, and which can be given without medical supervision, and do not require any pre-treatment screening test. Currently, means of studying the efficacy of drugs against relapsing malaria hypnozoites is limited. While it is possible to study drug efficacy against primate relapsing forms of malaria, there is no equivalent animal model available for *P. vivax*. While the *P. cynomolgi* primate model has a well demonstrated predictive value against *P. vivax* in humans [5], ultimately results from any *P. cynomolgi* trial and its applicability to human *P. vivax* infection can only be definitively assessed with a human trial.

Because of the uncertain applicability of animal models to human *P. vivax,* candidate drugs for vivax malaria may lack convincing evidence sufficient to justify an intensive trial that would divert resources from higher yield efforts and that would possibly expose an unacceptable number of subjects to a failing regimen if executed using a traditional study design. This study uses a sequential analysis method known as a modified triangular test in order to limit enrolment and exposure to the study product. This method has been used successfully with increasing frequency, including in a study of azithromycin against *P. vivax*[6-8].

The Military Infectious Diseases Research Program (MIDRP) has established development of new drugs as an objective. The ideal drug to prevent relapse would require a short course of treatment (five days or less), have an excellent safety profile, be non-haemolytic, have an accelerated path to formal approval and be widely available. In the alternative, a secondary, but still valuable drug would potentiate the effect of primaquine and allow for a lowering of its dose or shortening of its course thereby reducing haemolytic potential and increasing compliance. Tinidazole is a widely available approved 5-nitroimidazole drug used in the treatment of amoebiasis and giardiasis, that lacks significant P450 enzyme inhibition and has a proven safety profile [9,10]. *In vitro* testing with *Plasmodium falciparum* and mouse testing with *Plasmodium berghei* revealed no apparent blood stage activity and no or unacceptably slow blood stage activity with *Plasmodium cynomolgi* (unpublished observations Bennett K, 2007). Testing in a causal prophylaxis chick model with *Plasmodium gallinaceum* demonstrated increased survival from 4 days in control animals to 9.5 days in chicks treated with tinidazole (Walter Reed Army Institute of Research Chemical Information System Database 2008). Testing with ‘Rhesus’ macaques (*Macaca mulatta*) using a relapsing strain of *P. cynomolgi*, one of six monkeys was cured of liver stage infection in an arm with 7 daily 150 mg doses of tinidazole coupled with chloroquine to eliminate the blood stage of the infection, with the other five monkeys demonstrating a mild apparent delay to relapse [11]. Relapse in the macaque model was particularly long (30 and 68 days in two monkeys) *versus* controls (12 and 16 days in two monkeys), when dosed with chloroquine for blood stage treatment and a sub-therapeutic dose of primaquine. One open label human study in 30 subjects given 2 gms of tinidazole monotherapy once followed by weekly doses of 500 mgs for 10–12 months cleared all subjects of blood stage infection within 96 hours with no recurrences of *P. vivax*[12]*.* However, this report of tinidazole efficacy has significant limitations to include no control arm, conduct of the study in India where the strain of *P. vivax* reportedly has a low relapse frequency of about 10-20% [13], and with subjects receiving ongoing prophylactic dosing for 10–12 months with the study product tinidazole at 500 mg given weekly [12].

Overall previous studies suggested some liver stage activity in animals and man and possible potentiation of primaquine. However, the evidence was not sufficient to justify a full Phase II standard clinical trial on both scientific and ethical grounds. A clinical trial design was sought to further investigate tinidazole efficacy in preventing relapse of *P. vivax* that would limit enrolment to a failing regimen without discarding a potentially promising drug.

## Methods

### Subjects

Subjects were otherwise healthy adults, ethnic Karen/Burmese, non-pregnant, G6PD normal with *.P. vivax* infection. All were histidine-rich protein 2 (HRP-2) negative, reducing the likelihood of *P. falciparum* co-infection. Subjects were males and females who resided near Mae Sot, Thailand, along the Northwestern Thai/Burmese border. *Plasmodium vivax* in this region is generally chloroquine sensitive with a usual mean time to recurrence of 21 to 63 days if treated with chloroquine alone, which is the standard of care [14-17]. Primaquine for radical cure is not routinely used for persons living in this endemic area, due to toxicity risks and because re-infection is a continuous risk. Subjects were excluded if presenting with mixed malarial infection, anaemia or if there was any concomitant use of metronidazole, mebendazole or albendazole or use of any drug with known anti-malarial properties within the preceding four weeks prior to presentation.

### Study design

This was a randomized, open-label, unblinded study approved by the Walter Reed Army Institute of Research and the Faculty of Tropical Medicine Mahidol University institutional review boards conducted in accordance with International Conference on Harmonisation and Good Clinical Practice guidelines and applicable Department of Defense regulations. Written consent was obtained from all subjects in their native language. The study was conducted at the Shoklo Malaria Research Unit (Mae Kon Kin Clinic), Mae Sot, Thailand. Subjects were randomized to either a treatment arm with the potential of up to 60 subjects to receive 2 gms of tinidazole by mouth per day for 5 days coupled with oral chloroquine at 25 mg/kg/day for five days or a positive comparator arm of up to 24 subjects to receive primaquine by mouth 30 mg base per day for 14 days coupled with chloroquine at 25 mg/kg/day for five days. The treatment arm dose combination and regimen was chosen as it is already an approved treatment regimen for extraintestinal amoebiasis, and represented the anticipated maximal dose for anti-malarial administration. The positive comparator arm was added to confirm chloroquine susceptibility (expected 100% cure rate) and to obtain an estimate of the rate of *P. vivax* re-infection in this study population during the study period.

Randomization was done at a 2:1 ratio between the treatment and active comparator arms. Malaria blood smears were done daily until blood stage clearance was achieved for two consecutive days and then weekly follow- up smears were performed until Day 90 with a statistical cut-off of follow-up at Day 63. Primary endpoints were recurrence or lack of recurrence with *P. vivax* through Day 63 of follow-up. Subjects were followed for an additional month (to Day 90). This period of follow-up allowed us to capture essentially all early relapses that would occur under normal circumstances (within 63 days), as well as assess if tinidazole delayed but did not fully eliminate recurrence. Subjects without a recurrence at 90 days were considered to have achieved radical cure. Day 0 was defined as the first day a patient received a study drug. Subjects that presented with concomitant *P. falciparum* infection or were otherwise unable to complete the study were replaced with a new subject.

### Statistical analysis and assessment of efficacy

This study utilized a form of sequential analysis known as a modified triangular test in order to evaluate tinidazole efficacy in the prevention of *P. vivax* relapse. This method allows for repeated analysis as each subject reaches a study endpoint while maintaining a pre-specified α and β error. It allows enrolment to be stopped when there is information sufficient to reach a conclusion available from subjects that have already reached a study endpoint, but it requires the tested drug to demonstrate a substantial benefit to be considered successful. The study used straight line stopping boundaries with a closed continuation region. This means that while the number of total subjects needed was partially based on results as each subject reaches either the primary or secondary endpoint (cure at 63 days or relapse), the number of potential subjects was limited to 50 at which point one of the boundaries would have to be crossed. The mathematical basis for this technique has been explained in detail elsewhere [6,18,19].

In order to apply the modified triangular sequential analysis technique used in this study it is necessary to make a determination of thresholds for success and failure, to create statistical boundaries and then evaluate, via a pre-calculated formula after each subject reaches an endpoint, whether the success or failure threshold or boundary has been crossed or not.

Applied to this study, the key statistical end point is the proportion of subjects that did not have relapsing disease within the trial period. The goal was to determine whether that proportion is higher than the preset threshold value. That threshold is the largest response rate (absence of relapse) for which further investigation of tinidazole would be not worthwhile. In other words, it represents the point just beyond the maximum amount of failure that could be tolerated in order to suspend drawing a conclusion. Therefore, the null hypothesis is described as H_0_: *ρ > = ρ*_*0*_, where *ρ*_*0*_ represents the threshold value pre-determined beyond which tinidazole would be considered a failure and *ρ* represents the proportion actually observed in the study. Since the threshold value was pre-determined, the alternative hypothesis H_a_, was *ρ < ρ*_*a*_ where *ρ*_*a*_ represented the smallest response rate (no relapse) for which further investigations would be worthwhile. This can also be described as the smallest amount of success that was needed to warrant further investigation of tinidazole.

This specific study was designed to have a 5% type I error and a 90% power (α = 0.05 and β = 0.10). A relapse rate of <20% indicated an adequate response and thus the minimum improvement in relapse rate necessary to warrant further study at the β = 0.10 level, while a relapse rate > =45% indicated insufficient protection and response. Utilizing the description above, the *ρ*_*0*_ = 0.45 and the *ρ*_*a*_ = 0.20. With the use of these pre-determined parameters, the boundaries of the triangular test with specific Z and V statistics were determined based on the accumulation of data that precedes each discrete evaluation. Z is representative of the results to date prior to each calculation and V is representative of how many subjects had reached an endpoint at the time of each calculation.

The following information, based on the preset values was programmed into “R Version 2.7.2, 2008”, a free open-source statistical program created by the R Foundation for Statistical Computing (ISBN 3-900051-07-0) accessible from the R Project for Statistical Computing [20] as follows:

p0 < − 0.45

p1 < − 0.20

alpha < − 0.05

beta < − 0.10

Theta < − log ((p1* (1-p0))/(p0* (1-p1)))

A1 < − 2/Theta*log(1/(2*alpha))

A = A1-0.583*sqrt(p0*(1-p0))

Theta = Theta*(2*qnorm(1-alpha)/(qnorm(1-alpha) + qnorm(1-beta)))

Lamda = Theta1/4

As a result, the boundary lines for this investigation (demonstrated graphically with linear lines on a V versus Z graph) are represented by the formulas:

Lower boundary (values below which demonstrate significant improvement) Z = −4.17-0.33V

Upper boundary (values above which demonstrate no significant improvement) Z = 4.17-0.99V

During each analysis (each time a subject reached an endpoint), the Z and V values were determined by:

Z = S – N(p0)

V = N(p0)(1-p0)

Where p0 = 0.45

S = number of recurrences observed to that point and

N = the number of subjects included

**Figure 1:**
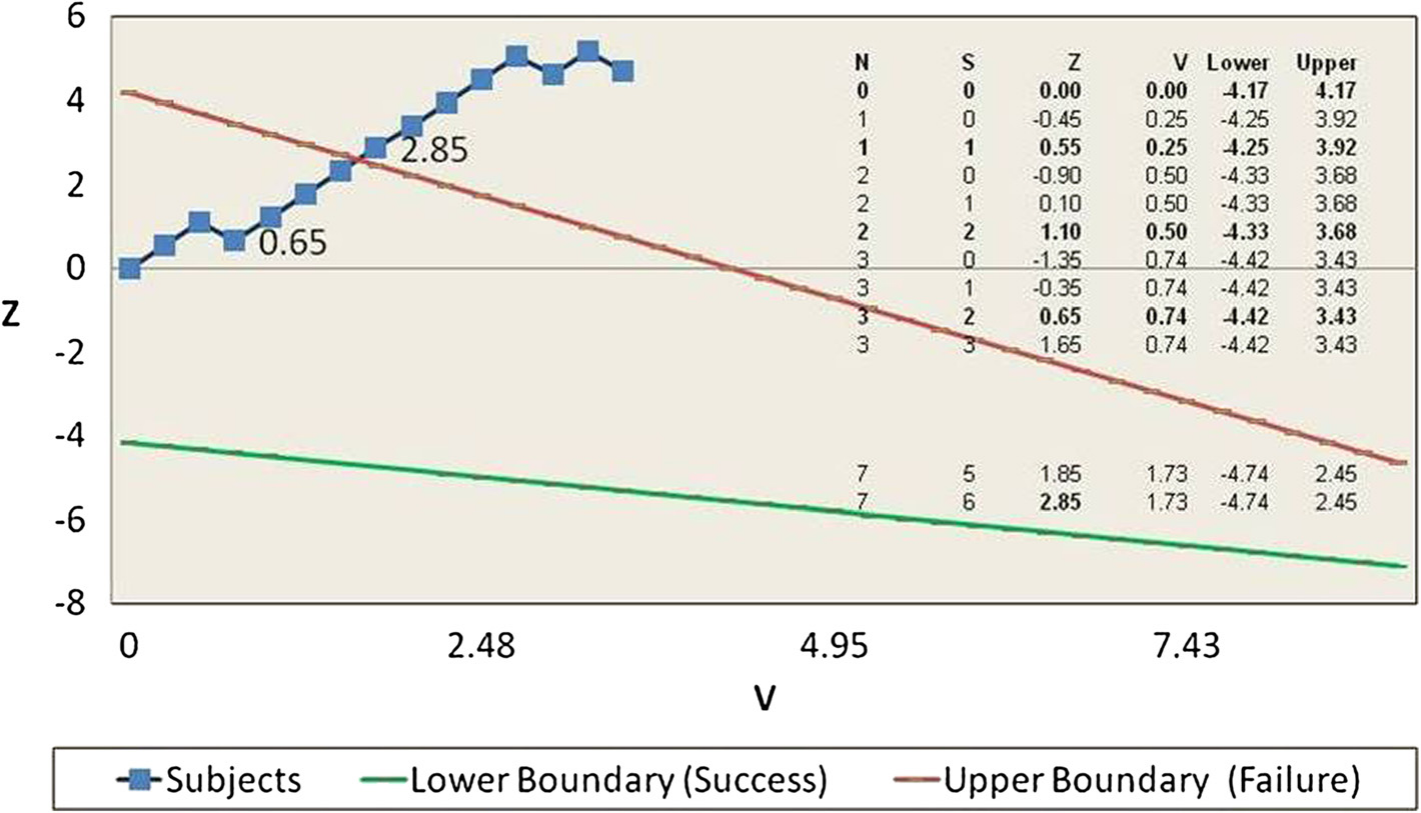
**legend text: Graphical representation of assessed Z and V scores and corresponding boundaries.** An evaluation was made after each subject reached an endpoint of recurrence prior to 63 days or non-recurrence at 63 days. Highlighted Z score values of 0.65 and 2.85 represent the third and seventh subject’s endpoint evaluation. Evaluation of the seventh subject crossed the failure threshold and enrolment was halted. Values after that point represent subjects already enrolled when enrolment was terminated.

In this investigation, an analysis was done after each subject either had a recurrence or reached Day 63 without a recurrence. With each analysis, a “V” and “Z” value (the x and y axes on a graphical plot in Figure 1) was calculated as well as the applicable limits of the test for that analysis (the boundary lines on a graphical plot). If the calculated “Z” value was above or below the Z value boundary line value, the study would be halted and the applicable conclusion made. If the calculated “Z” value was between the boundary values (within the triangular lines on the graphical plot), enrolment would continue unless 50 subjects had already reached the primary study endpoints of success at 63 Days (no recurrence) or failure (recurrence or co-infection) at which time the study would be stopped. While the actual determination of these values was pre-calculated for every possible permutation of results, they are best understood and illustrated by a graphical representation (See Figure 1) which also contains a partial listing of representative calculated values.

### Assessment of safety

A medical history and physical exam was performed at the time of screening. CBC (Complete blood count), ALT (alanine aminotransferase), GGT (gamma-glutamyl transpeptidase), BUN (Blood urea nitrogen), creatinine, glucose-6-phosphate-dehydrogenase (G6PD) levels, HRP-2 (Histidine-rich protein-2 specific to *P. falciparum* infection) and two thick/thin peripheral malaria blood smear slides were done to confirm diagnosis of *P. vivax* malaria mono-infection, and to quantify parasitaemia. Female subjects also submitted urine for a human beta chorionic gonadotropin (βHCG) test for pregnancy. The results of the screening history/physical and laboratory assessments were reviewed for any significant abnormality. Serum chemistries and complete blood counts were also done on Day 3 prior to the fourth dose of study drug and on Day 14. Subjects were monitored for adverse events and questioned about symptoms known to occur with use of the study drugs as well as for any other symptoms at each follow-up visit. While only those subjects reaching the primary endpoint of cure or the secondary endpoint of relapse were considered for efficacy analysis, all subjects enrolled receiving at least one dose of the study drug were included in all safety analyses.

## Results

20 active subjects were enrolled by the time the study crossed its statistical boundary of failure, 14 to the treatment arm and six to the positive comparator arm. In the treatment arm, 11 subjects had a recurrence of *P. vivax* within 63 Days and three subjects achieved cure of both liver and blood stages without relapse at day 90 (radical cure). Enrolment was halted when the 6th of the first seven subjects to reach an endpoint had a recurrence (Figure 1). In the positive comparator arm, all six subjects achieved radical cure and none were re-infected during the course of the study. Ten of the 11 failures in the treatment arm had an apparent mild delay in relapse with a mean of 47 days compared to the historical time to recurrence discussed above of approximately four weeks. While tinidazole may have prolonged the average time to relapse and resulted in occasional radical cure, it did not meet the pre-determined study threshold for success.

Three additionally enrolled subjects had to be replaced. One subject withdrew consent due to vomiting and difficulty swallowing pills. Another subject was removed from the study due to a mild elevation of his liver enzymes and slight jaundice and a third relocated from the region and was lost to follow-up. The drug combination was well tolerated. Adverse events were mild and mostly consisted of gastrointestinal complaints and headache.

## Discussion

The use of tinidazole in this study had the potential advantage of being a drug that is already approved for use in extra-intestinal amoebiasis in combination with chloroquine in the dose used in this trial. It also had the advantage of representing the possibility of a shorter dosing regimen than is the standard of care for achieving radical cure of *P. vivax* and without the potential of causing haemolysis. Unfortunately, tinidazole was unsuccessful in achieving radical cure with the dosing regimen used. However, several notable observations as a result of this study can be made.

The modest liver stage activity in *P. vivax* parallels results of the macaque/*P. cynomolgi* model with one of six cured and five of six achieving a mild delay in relapse with tinidazole, lending support to the use of the macaque/*P. cynomolgi* model in the evaluation of potential liver stage candidate drugs. Additionally, this study demonstrates the usefulness of a modified triangular test sequential analysis technique in limiting enrolment to a failing regimen, limiting exposure of the study population to the study drug and limiting expenditure with respect to products that have enough potential to warrant human study, but do not compel immediate full-scale investigation in a human model. This saves time, energy and resources that can be applied to other promising candidate drugs while at the same time not abandoning potential candidates prematurely that can only be truly studied in a human model.

## Conclusions

Tinidazole at 2 gms daily for 5 days given concurrently with chloroquine may show modest liver stage activity, but is ineffective in preventing the relapse of *P.vivax* malaria. The macaque relapsing model appears to accurately predict outcome for 5-nitroimidazoles in humans with symptomatic *P. vivax.* Use of the modified triangular test analysis allowed limited enrolment to a failing regimen adding weight to the ethical and economical advantages of this technique when appropriate.

## Competing interests

The authors declare that they have no competing interests.

## Authors’ contributions

LM drafted and defended the protocol, led the construction of study related documents, initiated the study and represented the sponsor in tracking study progress, wrote this manuscript and assisted in the construction of institutional agreements in order to conduct the trial. KML was the lead clinician enrolling subjects and implementing study procedures. PYC and PY assisted in the construction of study related documents and staff/site preparation for the study. SM acquired the funding for this trial and supervised trial design, site selection and the institutional agreements necessary to conduct the trial. FN was the principal investigator and director of the clinical trial site overseeing the conduct of the trial. All authors contributed to the final manuscript.
